# Cj0596 is a periplasmic peptidyl prolyl *cis*-*trans *isomerase involved in *Campylobacter jejuni *motility, invasion, and colonization

**DOI:** 10.1186/1471-2180-9-160

**Published:** 2009-08-08

**Authors:** Kimberly M Rathbun, Johanna E Hall, Stuart A Thompson

**Affiliations:** 1 Department of Biochemistry and Molecular Biology, Medical College of Georgia, Augusta, Georgia, USA

## Abstract

**Background:**

*Campylobacter jejuni *is a gastrointestinal pathogen of humans, but part of the normal flora of poultry, and therefore grows well at the respective body temperatures of 37°C and 42°C. Proteomic studies on temperature regulation in *C. jejuni *strain 81–176 revealed the upregulation at 37°C of Cj0596, a predicted periplasmic chaperone that is similar to proteins involved in outer membrane protein folding and virulence in other bacteria.

**Results:**

The *cj0596 *gene was highly conserved in 24 strains and species of *Campylobacter*, implying the importance of this gene. To study the role that Cj0596 plays in *C. jejuni *pathogenesis, a mutant derivative of strain 81–176 was constructed in which the *cj0596 *gene was precisely deleted. A revertant of this mutant was isolated by restoring the gene to its original chromosomal location using streptomycin counterselection. The *cj0596 *mutant strain demonstrated a slightly decreased growth rate and lower final growth yield, yet was more motile and more invasive of human intestinal epithelial cells than wild-type. In either single or mixed infections, the mutant was less able to colonize mice than 81–176. The *cj0596 *mutant also expressed altered levels of several proteins.

**Conclusion:**

Mutation of *cj0596 *has an effect on phenotypes related to *C. jejuni *pathogenesis, probably due to its role in the proper folding of critical outer membrane proteins.

## Background

*Campylobacter jejuni *is a curved, microaerophilic Gram-negative bacterium that is an important human pathogen [1,2]. The main reservoir of *C. jejuni *is the cecum of poultry, and humans can become infected through consumption of undercooked chicken that is contaminated with the bacterium [2]. In humans, the bacterium colonizes both the small and large intestine resulting in fever, severe abdominal pain, and diarrhea, with possible autoimmune sequelae of infection including Guillain-Barré syndrome and reactive arthritis [3].

Expression of proteins is an energetically costly activity. Therefore, bacteria often express certain proteins only under conditions where those proteins are needed for growth, survival, or pathogenicity. Growth temperatures cause differential expression of proteins in a number of pathogenic bacteria including *C. jejuni *[4-13], although the mechanisms of thermoregulation can be complex and result from overlapping regulatory systems. In *C. jejuni*, the RacRS two-component regulatory system is involved in the regulation of some proteins, although the majority of the targets have not been identified [14]. Because the body temperatures of humans and chickens differ (37°C and 42°C, respectively), *C. jejuni *is likely to express different proteins when colonizing chickens than when colonizing humans. We used proteomics to determine which *C. jejuni *proteins are more highly expressed at 37°C compared to 42°C, because such upregulation might suggest an importance of these proteins in colonization of humans. One of the proteins identified was Cj0596, which is annotated as playing a role in outer membrane protein folding and stabilization.

Cj0596 was previously identified as an immunogenic outer membrane protein and was named cell binding factor 2 (cbf2); it is also called PEB4 [15-19]. It was later suggested that PEB4 is not surface exposed, but is periplasmically located in association with the inner membrane [16]. Cj0596 shows homology to SurA, a peptidyl-prolyl *cis-trans *isomerase (PPIase) found in *E. coli*, and other orthologs in numerous bacteria including *Helicobacter pylori*, *Bacilis subtilis*, and *Lactococcus lactis *[20-22]. PPIases have been characterized as virulence factors in *Shigella flexneri*, *Salmonella enterica*, *Legionella pneumophila*, *Chlamydia trachomatis*, *Trypanosoma cruzi*, and *Neisseria gonorrhoeae *[23-28]. Asakura *et al*. [29] recently characterized a *cj0596 *mutant of *C. jejuni *strain NCTC 11168, finding decreases in ability to adhere to INT407 cells and to colonize mice, and an increase in biofilm formation. However, this mutant was not complemented with a wild-type copy of *cj0596*, allowing some question of whether the observed phenotypes were specific for Cj0596.

In this study, we examine the effects of deletion of *cj0596 *in a different, highly invasive *C. jejuni *strain (81–176) on phenotypes related to growth, protein expression, and pathogenicity. Our results show that deletion of *cj0596 *alters several virulence-related phenotypes *in vitro *and decreases the ability of the bacterium to colonize mice, although the phenotypes of the 81–176 *cj0596 *mutant differ significantly from those of an analogous mutant in strain NCTC 11168.

## Methods

### Bacterial strains and routine culture conditions

*Campylobacter jejuni *strains derived from the parent 81–176 [30,31] (Table 1) were routinely maintained with minimal passage on blood agar plates (Remel; Lenexa, KS) at 37°C in sealed culture boxes (Mitsubishi Gas Chemical [MGC], New York, NY) containing a microaerobic atmosphere generated by Pack-Micro Aero (MGC). Liquid cultures of *C. jejuni *were grown in Brucella broth or Mueller-Hinton (MH) broth and cultured in microaerobic environments. When appropriate, strains were cultured in the presence of chloramphenicol (30 μg/ml) or streptomycin (30 μg/ml) to select for antibiotic resistance markers.

**Table 1:** Strains used in this study

Strain	Reference or source
*C. jejuni *81–176	[30]

*C. jejuni *81–176*cj0596*	This study

*C. jejuni *81–176*cj0596*^+^	This study

*C. jejuni *NCTC11168	[22]

*C. jejuni *81116	[43]

*C. jejuni *HB95-29	[44]

*C. jejuni *INP44	[44]

*C. jejuni *INP59	[44]

*C. coli *D3088	[44]

*C. jejuni *RM1221	TIGR CMR [62]

*C. jejuni *subsp. *doylei *269.97	TIGR CMR [62]

*C. jejuni *subsp. *jejuni *260.94	TIGR CMR [62]

*C. jejuni *subsp. *jejuni *84-25	TIGR CMR [62]

*C. jejuni *subsp. *jejuni *CF93-6	TIGR CMR [62]

*C. jejuni *subsp. *jejuni *CG8486	[45]

*C. jejuni *subsp. *jejuni *HB93-13	TIGR CMR [62]

*C. coli *RM2228	TIGR CMR [62]

*C. concisus *13826	TIGR CMR [62]

*C. curvus *525.92	TIGR CMR [62]

*C. fetus *subsp. *fetus *82–40	TIGR CMR [62]

*C. hominis *ATCC BAA-381	TIGR CMR [62]

*C. lari *RM2100	TIGR CMR [62]

*C. upsaliensis *RM3195	TIGR CMR [62]

*E. coli *BL21(DE3)pLysS	[32]

*H. pylori *84–183	[50]

*Escherichia coli *JM109 was used as the host strain for cloning experiments and *E. coli *BL21(DE3)pLysS [32] was used as the host strain for expression of the his-tagged Cj0596 protein. *E. coli *strains were cultured in Luria-Bertani (LB) broth or agar [33], supplemented with the following antibiotics as appropriate for selection of plasmids: ampicillin, 50 μg/ml; chloramphenicol, 30 μg/ml; streptomycin, 30 μg/ml.

### Proteome analysis of *C. jejuni *strains

Proteomics experiments were performed on *C. jejuni *cells grown at 37°C and 42°C as described [34]. Briefly, cells were grown overnight at 37°C in Brucella broth, then diluted the following morning into two aliquots of fresh Brucella broth (OD_600 _= 0.1), which were grown at 37°C and 42°C to mid-log phase (OD_600 _= 0.1). Chloramphenicol (187 μg/ml) was added to stop protein synthesis [35], and the cells were harvested for proteome analysis as described [34]. Proteomics experiments were performed using Differential In-Gel Electrophoresis (DIGE) technology from GE Biosystems (Piscataway, NJ), Whole-cell protein lysates from the 37°C- and 42°C-grown *C. jejuni *(25 μg each) were labelled individually with Cy3 and Cy5 dyes according to the protocol supplied by the manufacturer (GE Biosystems), then mixed in equal mass and separated using two-dimensional (2D) SDS-PAGE.

For comparison of WT, mutant and revertant proteomes (see below), protein lysates (25 μg each) were labelled with Cy2 (wild-type 81–176), Cy3 (mutant 81–176*cj0596*), or Cy5 (revertant 81–176 *cj0596*^+^) dyes, mixed in equal mass, and separated by 2D SDS-PAGE.

Following separation, gels were scanned on a Typhoon fluorescent flatbed scanner (GE Biosystems), at the following wavelengths: Cy2, 488 nm excitation, 520 nm emission, Cy3, 532 nm excitation, 580 nm emission; Cy5, 633 nm excitation, 670 nm emission. Images were analyzed with Decyder Differential In-Gel Analysis (DIA) software (version 4.0, GE Biosystems) for identification of proteins with higher or lower expression in different samples. The identities of proteins of interest were determined using a matrix-assisted laser desorption ionization – time-of-flight/time-of-flight (MALDI-ToF/ToF) spectrometer (Applied Biosystems, Foster City, CA), using both tryptic fingerprint data and fragmentation-based MS/MS.

### Purification of Cj0596 protein and antibody production

To allow purification of the Cj0596 protein, a C-terminal his_6_-tag was added to *cj0596 *lacking the N-terminal signal sequence by inserting the gene into pET-20b(+). First, the *cj0596 *gene without the signal sequence and stop codon was amplified from *C. jejuni *strain 81–176 and Nde I and Xho I sites were added using primers purprot-F and purprot-R (Table 2). The resulting PCR product was cloned into pCR II-TOPO, creating plasmid pKR016 (Table 3). Using Nde I and Xho I, the *cj0596 *gene was excised from pKR016 and pET-20b(+) was linearized. The *cj0596 *gene was ligated into the linearized pET-20b(+) creating plasmid pKR017, which was used to transform *E. coli *strain BL21(DE3)pLysS (Table 1). The plasmid-carrying strain was grown overnight in LB broth at 37°C. The next morning the culture was diluted to OD_600 _~ 0.1 and incubated at 37°C until OD_600 _~ 0.5. IPTG was added to the culture to induce expression of the his_6_-tagged protein. After 2 h, the cells were harvested by centrifugation, washed, and the supernatant passed through a nickel column to further purify the his_6_-tagged protein by standard methods [36]. The purified protein was sent to Cocalico Biologicals, Inc. (Reamstown, PA) for production of anti-Cj0596 antibodies. For use in the PPIase assay, the protein was refolded using the Pro-Matrix Protein Refolding Kit (Pierce Biotechnology, Inc.) and dialyzed against PBS.

**Table 2:** Primers used in this study

Primer	**Sequence (5' → 3')**^†^
purprot-F	**CATATG**GCTACAGTAGCTACTGTT

purprot-R	**CTCGAG**TTTATATTCCACTTT

*cj0596*-F1	TTTTAGCTTTACAGGTGTAACG

*cj0596*-R1	ATCCACTCCATCTTCTTCGC

*cj0596*-inv1	**ACCGGT**TTTTAATAAAATGTATATAA

*cj0596*-inv2	**ACCGGT**TAT**GCTAGC**AAAGTGGAATATAAATAATGGG

*cat*-F1	**ACCGGT**AAAA**CAATTG**GGAGGATAAATGATGCAATT

*cat*-R1	**GCTAGC**TTATTTATTCAGCAAGTCTT

*rpsL*_*HP*_-F1	**ACCGGT**AACGACTAAAGTTTTAACA

*rpsL*_*HP*_-R1	**CAATTG**TCTTCAATCTTATTAAAAGCT

*cj0597*RT-F	GACACAGATACTCAAATGGGC

*cj0597*RT-R	CCAAACACGCGGATCATA

16S-RT-F	GGGTGCTAGTCATCTCAGTAATGC

16S-RT-R	GGTAAGGTTCTTCGCGTATCTTCG

purprot-F	**CATATG**GCTACAGTAGCTACTGTT

purprot-R	**CTCGAG**TTTATATTCCACTTT

^†^Bold nucleotides indicate restriction sites introduced for cloning purposes

**Table 3:** Plasmids used in this study

Plasmid	Description	Source
pET-20b(+)	Cloning vector, Amp^R^	Novagen

pCR II-TOPO	Cloning vector, Kan^R ^Amp^R^	Invitrogen

pKR001	*cj0596 *gene and flanking region in pCR II-TOPO	This study

pKR002	Self-ligated inverse PCR product of pKR001 with *cj0596 *removed	This study

pKR016	*cj0596 *gene lacking signal sequence and stop codon in pCR II-TOPO	This study

pKR017	*cj0596 *gene lacking signal sequence and stop codon in pET-20b(+)	This study

pKR018	*cat *cassette in pCR II-TOPO	This study

pKR019	*H. pylori *84–183 *rpsL *gene in pCR II-TOPO	This study

pKR020	*cat *cassette in pKR002	This study

pKR021	*rpsL*_*HP*_/*cat *construct in pKR002	This study

To confirm the proteomics-implicated temperature regulation of Cj0596, a western blot was performed on *C. jejuni *cells grown at 37°C or 42°C using anti-Cj0596 antibody (1:1,000) as the primary antibody and HRP conjugated goat anti-rabbit IgG (1:50,000) as the secondary antibody. A control western blot against Cj0355 (expression of which is unaffected by growth temperature (Fields and Thompson, unpublished results; [37]) was performed using anti-Cj0355 antibody (1:1,000) as the primary antibody and HRP conjugated goat anti-rabbit IgG (1:50,000) as the secondary antibody. The blots were developed using a DAB Substrate Kit (BD Biosciences). Densitometry measurements were conducted using ImageJ software [38].

### Localization of the Cj0596 Protein

To determine the cellular location of Cj0596, *C. jejuni *cells grown at 37°C were separated into cytoplasmic, periplasmic, and inner membrane fractions [39], and outer membrane fractions as described [37]. Western blots were performed on *C. jejuni *cell fractions using anti-Cj0596 antibody (1:1,000) as the primary antibody, along with control blots using anti-Cj0355 (cytoplasmic control), anti-CetA (inner membrane control), and anti-MOMP (outer membrane control) polyclonal sera (all at 1:1,000) as the primary antibodies. HRP conjugated goat anti-rabbit IgG (1:50,000) was used as the secondary antibody for all blots, which were then developed using a DAB Substrate Kit (BD Biosciences).

### PPIase Assay

The PPIase activity of Cj0596 was determined in a coupled assay, which measures the ability of Cj0596 to convert the *cis *isomer of the oligopeptide substrate *N*-Suc-Ala-Ala-Pro-Phe-*p*-nitroanilide into the *trans *form which is cleavable by α-chymotrypsin [40-42]. Chymotrypsin (0.63 μg/ml) and varying concentrations of Cj0596 were combined in 50 mM Tris-HCl pH 7.8 and incubated at 4°C. The substrate (93.8 μg/ml) was added and the reaction was monitored at 10°C by the increase in absorbance at 390 nm (corresponding to the release of *p*-nitroanilide). The k_obs _value for each PPIase concentration was found by plotting Ln [A_390_(∞)-A_390_(t)] vs. time (sec) and determining the slope. The catalytic efficiency (*k*_cat_/*K*_m_) of the PPIase activity was obtained by plotting k_obs _vs. [PPIase] and determining the slope.

### Analysis of *cj0596 *in additional *Campylobacter *strains

The DNA sequences of the *cj0596 *locus (gene designation from strain NCTC 11168; the strain 81–176 designation is CJJ81176_0624) were determined from the *Campylobacter *strains listed in Table 1[22,30,43-45]. Primers *cj0596*-F1 and *cj0596*-R1 (Table 2) were designed based on the published NCTC 11168 genome sequence and used in PCR reactions with template genomic DNA prepared using a MasterPure DNA purification kit (Epicentre, Madison, WI) to amplify the region encompassing *cj0596*. Thermocycler parameters were 35 cycles of: 94°C for 30 seconds, 51°C for 30 seconds, and 72°C for 4 minutes. PCR products were purified using QIAquick PCR purification kits (Qiagen, Valencia, CA) and were then sequenced directly (both strands), using an ABI 3730xl sequencer (Applied Biosystems). VectorNTI (version 7, Invitrogen, Carlsbad, CA) was used to analyze DNA sequences. The protein sequences were analyzed for motifs using the ExPASy Prosite server http://au.expasy.org/prosite/[46,47], and potential signal peptides were evaluated using SignalP 3.0 http://www.cbs.dtu.dk/services/SignalP/[48].

### Isolation of a *C. jejuni cj0596 *mutant

A *cj0596 *mutant of *C. jejuni *strain 81–176 was constructed using a streptomycin counterselection system similar to the heterologous *H. pylori*-*C. jejuni *method described by Dailidiene *et al*. [49] in which an *rpsL *gene from *C. jejuni *was used for counterselection in *H. pylori*, to decrease background gene conversion events. In the heterologous *rpsL*_*HP *_system reported here, *cj0596 *was exactly replaced by the *rpsL*_*HP *_(Str^S^) gene from *H. pylori *strain 84–183 (Table 1; [50]) linked to a chloramphenicol acetyltransferase (*cat*) cassette (Cm^R^). This strategy allows for selection of a mutant (Str^S^/Cm^R^) based on chloramphenicol resistance and then allows for selection of a revertant strain (Str^R^/Cm^S^) based on streptomycin resistance. First, a PCR-amplified *cj0596 *gene was amplified using primers *cj0596*-F1 and *cj0596*-R1 designed based on the published *C. jejuni *NCTC 11168 genome sequence (Table 2) and the resulting product was cloned into pCR II-TOPO creating pKR001 (Table 3). The plasmid pKR001 was subjected to inverse PCR (primers *cj0596*-inv1 and *cj0596*-inv2) to remove the *cj0596 *gene and add restriction sites. The inverse PCR product was self-ligated to form plasmid pKR002. The *cat *cassette was amplified from pRY111 [51] and Age I, Mfe I, and Nhe I sites were added using primers *cat*-F1 and *cat*-R1. The resulting PCR product was cloned into pCR II-TOPO to create plasmid pKR018. The *rpsL*_*HP *_gene was amplified and Age I and Mfe I sites were added using primers *rpsL*_*HP*_-F1 and *rps*L_HP_-R1 and the PCR product was cloned into pCR II-TOPO creating plasmid pKR019. Age I and Nhe I were used to excise the *cat *cassette from pKR018 and linearize pKR002. The *cat *cassette was ligated into pKR002 creating plasmid pKR020. Using Age I and Mfe I, the *rpsL*_*HP *_gene was removed from pKR019 and ligated with linearized pKR020, creating plasmid pKR021, with the *rpsL*_*HP*_/*cat *construct oriented in the same direction as the *cj0596 *and *cj0597 *genes. Care was taken to ensure that this replacement would not produce polar effects by preserving the *cj0597 *ribosome binding site and by leaving only 23 bp between the stop codon of *cat *and the start codon of *cj0597*. The mutagenized *cj0596 *allele was introduced into a spontaneous Strep^R ^derivative of *C. jejuni *81–176 by electroporation. Several Cm^R^/Strep^S ^transformants were verified as *cj0596 *mutants by PCR with primers *cj0596*-F1 and *cj0596*-R1 (Table 2) and DNA sequencing (data not shown), a representative of which was designated 81–176*cj0596 *(Table 1) and used for further analysis.

### Reversion of the *cj0596 *mutation

A revertant of *C. jejuni *81–176*cj0596 *was isolated by replacing the mutated *cj0596 *allele in 81–176*cj0596 *with a wild-type *cj0596 *gene using streptomycin counterselection. *C. jejuni *strain 81–176*cj0596*^+ ^was created by using electroporation to introduce pKR001 into 81–176*cj0596 *cells, selecting for colonies on plates containing streptomycin (30 μg/ml). Putative revertants were identified by screening Str^R ^colonies for sensitivity to chloramphenicol (30 μg/ml) to ensure loss of the *rpsL*_*HP*_/*cat *cassette. Chromosomal DNA was isolated from these transformants and proper replacement of the *rpsL*_*HP*_/*cat *cassette with wild-type *cj0596 *was confirmed by PCR using primers *cj0596*-F1 and *cj0596*-R1 (Table 2) and by DNA sequencing of the region.

### Quantitative real-time reverse transcription PCR

cDNA was prepared from RNA samples of *C. jejuni *grown 81–176 and 81–176*cj0596 *using a GeneAmp RNA PCR kit (Applied Biosystems). An ICycler IQ real-time PCR detection system (Bio-Rad Laboratories, Hercules, CA) was used to run qRT-PCR with IQ Sybr Green Super Mix, and primers *cj0597*RT-F and *cj0597*RT-R (Table 2). Data were analyzed using Bio-Rad ICycler data analysis software. Control reactions used primers specific for 16S rDNA (16S-RT-F and 16S-RT-R, Table 2), which allows amplification of a non-regulated RNA [52]. Differences in transcript levels among samples were calculated from amplification profiles using the comparative threshold cycle (ΔΔCT) method, as previously described [53].

### Growth experiments

The growth rates of *C. jejuni *wild-type 81–176, mutant 81–176*cj0596*, and revertant 81–176*cj0596*^+ ^were assessed by growing cells overnight in MH broth, then diluting the following morning in MH Broth to OD_600 _~ 0.06 (the *cj0596 *mutant was additionally inoculated at OD_600 _~ 0.2 due to poor correlation between OD_600 _and CFU for this strain; see Results) and shaking at 37°C under microaerobic conditions. Growth was monitored by measuring OD_600 _and numbers of viable bacteria were determined by plating serial dilutions of the bacterial suspensions on MH agar and counting the resultant colonies.

### Motility

The motility of *C. jejuni *81–176, 81–176*cj0596*, and 81–176*cj0596*^+ ^was determined as previously described [54]. Briefly, cells were grown overnight in MH broth, then diluted the following morning in MH broth to OD_600 _= 0.1. A pipet tip dipped into the suspension was used to stab the center of a MH motility plate (0.4% agar). The plates were incubated at 37°C and the diameter of the motility zone was measured every 12 h.

### Adherence/Invasion/Intracellular survival assay

A gentamicin protection assay [34,55] was used to assess the ability of 81–176, 81–176*cj0596*, and 81–176*cj0596*^+ ^to adhere to, invade, and survive within INT407 human intestinal epithelial cells. Briefly, bacteria were grown in biphasic [brain heart infusion (BHI)/1% yeast extract (YE)] cultures at 37°C under microaerobic conditions for ~20 h. Bacteria were harvested, resuspended in phosphate buffered saline (PBS), then added in triplicate to semi-confluent INT407 cell monolayers (~1 × 10^5 ^cells/well) at a multiplicity of infection (MOI) of ~40:1 (bacteria:epithelial cells). The number of bacteria added was quantified by determination of CFU/mL. The cells were incubated for 3 h at 37°C under microaerobic conditions and were washed with Hanks' Balanced Salt Solution (HBSS), lysed with Triton X-100 and the number of adherent bacteria was quantified by viable counts. For determination of invasion, cells were incubated for 3 h with bacteria and then gentamicin was added to a final concentration of 250 μg/ml to kill any extracellular bacteria. After an additional 2 h of incubation, the cells were washed, lysed with Triton X-100 and intracellular bacteria were quantified by viable counts. The gentamicin and Triton X-100 MICs of the three strains were also determined.

For determination of intracellular survival, the cells were incubated for 3 h with bacteria, 2 h with gentamicin, and then the INT407 cells were washed and incubated for 4 h in minimal essential media containing 3% fetal bovine serum and gentamicin (10 μg/ml) as described by Candon *et al*. [56]. After the incubation period, cells were washed and lysed with Triton-X 100 and the number of bacteria that survived intracellularly was quantified by viable counts.

### Mouse Colonization Experiments

The *in vivo *relevance of Cj0596 was investigated by testing the ability of 81–176, 81–176*cj0596*, and 81–176*cj0596*^+ ^to colonize mice as described [34,57,58]. 10-week old female BALB/c-ByJ mice were given 500 μl of 5% sodium bicarbonate by oral gavage to neutralize stomach acid. The mice were then given a dose of 1 × 10^9 ^CFU in 500 ml of BHI/1% YE broth by oral gavage. Because there was an observed discrepancy between OD_600 _and CFU for the mutant (see Results), we first performed pilot experiments correlating OD_600 _and CFU for all of the strains. After four repetitions, we found the mutant OD_600 _that gave the same number of CFU as for the WT and revertant strain, and this is what we used for the mouse inocula. We also verified that each mouse received equal CFU by plating the inocula for viable counts at the time of inoculation. Colonization was determined by viable counts of bacteria in fecal pellets, which were enumerated on selective CVA media [59] starting 7 days after the challenge and continuing every 7 days thereafter until 35 days post-inoculation.

In parallel, experiments were carried out to determine the ability of *cj0596 *mutant bacteria to compete with wild-type bacteria in colonization. For competition experiments, wild-type and mutant bacteria were mixed in equal amounts (5 × 10^8 ^CFU each) immediately prior to inoculation. Colonization was determined by enumerating bacteria on selective media with or without chloramphenicol (30 μg/ml). The number of bacteria counted on the plates containing chloramphenicol (viable mutant bacteria) was subtracted from the number of bacteria found on the plates without chloramphenicol (total of mutant and wild-type bacteria) to obtain the number of viable wild-type bacteria. Control experiments showed that the plating efficiency of the Cj0596 mutant was equivalent on media containing or lacking chloramphenicol. All vertebrate animal experiments were conducted in accordance with recommendations by the Office of Laboratory Animal Welfare, and were approved by the Medical College of Georgia Institutional Animal Care and Use Committee (MCG IACUC; protocol 04-03-379B, approved 3/18/2004).

## Results

### Expression of *cj0596 *is slightly higher at 37°C than at 42°C

**Figure 1:**
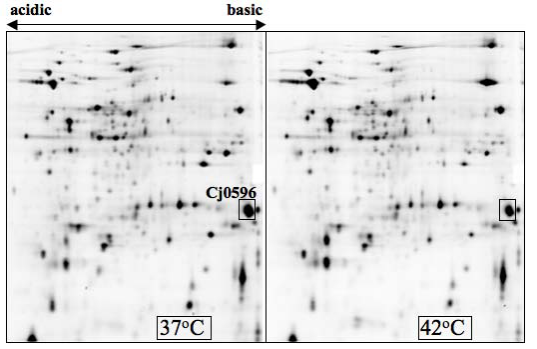
**Temperature-dependent changes in the expression level of the Cj0596 protein**. Two-dimensional SDS-PAGE protein gel showing the expression of *C. jejuni *81–176 proteins at 37°C and 42°C. The Cj0596 protein identified using mass spectrometry is indicated by a box.

In a search to identify *C. jejuni *genes with differential response to steady-state growth temperature (37°C vs. 42°C), several proteins were identified that were more highly expressed at 37°C than at 42°C. *C. jejuni *81–176 was grown overnight at 37°C and then diluted into fresh media. The two cultures were grown in parallel at 37°C and 42°C to mid-log growth phase. Proteomics experiments were then performed on cultures of *C. jejuni *81–176 grown at the two temperatures. One protein that was upregulated at 37°C had the approximate pI and molecular mass of the predicted Cj0596 protein (Figure 1). This protein was 1.8-fold more highly expressed at 37°C, a result that was consistent in five different proteomics experiments. The protein was excised from the polyacrylamide gel and subjected to MALDI-ToF/ToF mass spectrometry. This protein was identified with 100% confidence as Cj0596 (data not shown).

In an attempt to confirm the proteomics results, we performed western blots using anti-Cj0596 antibodies and *C. jejuni *81–176 grown at 37°C and 42°C. While only semi-quantitative, in two separate experiments the western blots showed a more modest 1.3–1.6-fold greater expression of Cj0596 at 37°C (data not shown). Therefore, we conclude that while Cj0596 appears to be expressed at a somewhat higher level at 37°C compared to 42°C, the effect of growth temperature on Cj0596 expression is not dramatic. Because proteins homologous to Cj0596 are involved in virulence in other pathogenic bacteria, we nevertheless characterized the role of this protein in *C. jejuni *physiology and pathogenesis.

### Similarity of *cj0596 *sequences among *Campylobacter *species

**Figure 2:**
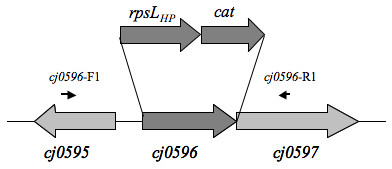
**Construction of a *cj0596 *mutant in *C. jejuni *81–176**. The location of the replacement of the *cj0596 *gene by the *rpsL*_*HP*_/*cat *construct is shown. Solid arrows represent PCR primers used to amplify the *cj0596 *region during mutant construction and verification, and for interstrain comparative DNA sequencing.

Because *Campylobacter *genomes are quite diverse [60,61], we characterized the conservation of the *cj0596 *gene in other *Campylobacter *strains. Using PCR primers designed from the *C. jejuni *NCTC 11168 genomic sequence and located in the *cj0595 *and *cj0597 *genes (Figure 2), we amplified a 2 kb segment encompassing the *cj0596 *locus from five additional *C. jejuni *strains and one *C. coli *strain. PCR products of the expected size were obtained from each strain, and were subsequently sequenced (total of 4000 bp sequence analyzed for each strain). A search of 17 additional *Campylobacter *genome sequences (Table 1) was also performed and showed that a *cj0596 *ortholog was found in every strain. The sequences of these orthologs were also included in the sequence comparison analysis. The nucleotide sequences between pairs of *C. jejuni *strains or *C. coli *D3088 were at least 98% identical. The corresponding sequences from *C. coli *RM2228 and other *Campylobacter *species were somewhat lower (84% to 60% identical). The predicted Cj0596 protein was also highly similar in all *C. jejuni *strains and *C. coli *D3088, with an amino acid sequence identity of at least 99%. As with the nucleotide sequences, the degree of identity of proteins from *C. coli *RM2228 and other non-*jejuni Campylobacter *strains was lower, with identities ranging from 87% to 45%. Together, these results indicate that *cj0596 *is highly conserved in *C. jejuni *(16 strains), *C. coli *(two strains), and one strain each of *C. concisus*, *C. curvus, C. fetus*, *C. hominis*, *C. lari*, and *C. upsaliensis*. We focused on Cj0596 from *C. jejuni *strain 81–176 (the strain 81–176 designation is CJJ81176_0624) for our subsequent work.

### *In silico *analysis of Cj0596 protein features

In the NCTC 11168 genome, the predicted Cj0596 protein had a predicted molecular mass of 30.5 kDa and pI of 9.9 and was annotated as a major antigenic peptide PEB4\cell binding factor 2, similar to peptidyl prolyl *cis-trans *isomerases found in a variety of organisms [62]. Because some peptidyl-prolyl *cis-trans *isomerases are located in the periplasm, the SignalP algorithm [48,63] was used to analyze the 81–176 Cj0596 protein for the presence of an N-terminal signal sequence. A signal sequence with a probable cleavage site between amino acids 21 and 22 of the preprotein (VNA↓AT) was predicted. Cj0596 was also analyzed for the presence of protein motifs [46] and was found to contain a PpiC-type peptidyl-prolyl *cis-trans *isomerase motif located between amino acids 131–228. Secondary structure predictions showed no transmembrane segments in the mature protein, suggesting that Cj0596 is likely to be a periplasmic protein.

### Cj0596 is located in the periplasm of *C. jejuni*

**Figure 3:**
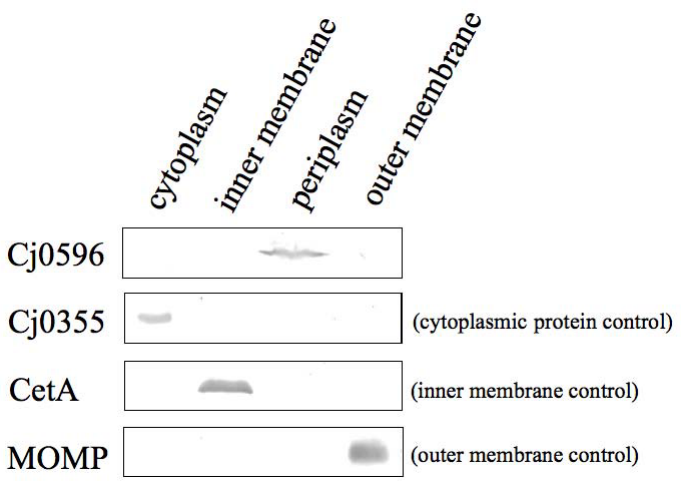
**Localization of Cj0596**. Western blots of cell fractions using Cj0355 as a cytoplasmic control, CetA as an inner membrane control, and MOMP as an outer membrane control show that Cj0596 is located in the periplasm of *C. jejuni*.

The amino acid sequence of Cj0596 suggested that this protein is located in the periplasm. To test this experimentally, western blots were performed on cytoplasmic, inner membrane, periplasmic, and outer membrane fractions of *C. jejuni *81–176 using anti-Cj0596, anti-Cj0355, anti-CetA, and anti-MOMP antibodies (Figure 3). As expected, anti-Cj0355 antibodies reacted with a ~25 kDa protein in the cytoplasmic fraction, anti-CetA antibodies reacted with a ~50 kDa protein in the inner membrane fraction, and anti-MOMP antibodies reacted with a ~45 kDa protein in the outer membrane. Anti-Cj0596 antibodies reacted with a ~30 kDa protein present primarily in the periplasmic fraction.

### Cj0596 has PPIase Activity

**Figure 4:**
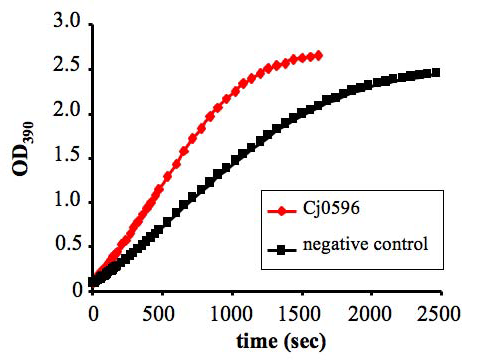
**PPIase activity of Cj0596**. Enzymatic activity of Cj0596 assayed by cleavage of N-Suc-Ala-Ala-Pro-Phe-*p*-nitroanilide in a chymotrypsin-coupled assay, in which cleavage of the *trans *isomer of the substrate by chymotrypsin is accelerated by PPIase activity. A representative plot of Absorbance vs. time using purified Cj0596 protein (red diamonds) and negative control (black squares) is shown.

Cj0596 has one rotamase domain and is similar to *E. coli *SurA, suggesting that it is a PPIase. The PPIase activity of purified Cj0596 was determined using a coupled assay in which the cleavage of the *trans *isomer of *N*-Suc-Ala-Ala-Pro-Phe-*p*-nitroanilide by α-chymotrypsin results in the release of *p*-nitroanilide, causing a colorimetric change over time. Conversion of the *cis *to the *trans *isomers of the substrate occurs spontaneously in solution, allowing chymotrypsin cleavage (Figure 4, squares). However, addition of Cj0596 accelerates this *cis-trans *conversion, indicative of PPIase activity (Figure 4, diamonds). By using varying concentrations of purified Cj0596 (data not shown) and plotting calculated k_obs _vs. [PPIase], the PPIase activity (k_cat_/k_m_) was calculated to be 22.3 mM^-1^sec^-1^, an activity consistent with values published for other PPIases [64-66].

### Creation of a non-polar *cj0596 *mutation

To test the role of Cj0596 in *C. jejuni *physiology or pathogenesis, we created a non-polar *cj0596 *mutant. To facilitate mutant construction, we developed a modified streptomycin counter selection system based on a similar strategy used in *H. pylori *[49]. The *rpsl*_*HP*_/*cat *cassette (Methods) was used to precisely replace *cj0596*, maintaining the ribosome binding site of the downstream *cj0597 *gene. Together with the lack of a transcriptional terminator on the *rpsl*_*HP*_/*cat *cassette, this construct is expected to yield a non-polar mutation. The resulting mutant strain was designated 81–176*cj0596*. To confirm that mutation of the *cj0596 *gene did not alter the expression of the putative co-transcribed *cj059*7 gene, quantitative real-time RT-PCR was performed on RNA samples harvested from 81–176 and 81–176*cj059*6. These studies confirmed that mRNA levels downstream of the mutation in 81–176*cj0596 *were equivalent to those in 81–176, and that the mutation was non-polar (data not shown). However, to ensure that any phenotypes of the *cj0596 *mutant were specific for *cj0596*, we subsequently isolated a reversion of the mutation by replacing the *rpsl*_*HP*_/*cat *cassette with a wild-type *cj0596 *gene. Analogous to the counterselection system of Dailidiene et al. [49], use of the *H. pylori rpsL *gene decreased background gene conversion events and facilitated the recovery of cells in which the mutagenized *cj0596 *allele was replaced with the wild-type *cj0596 *gene. This process was extremely efficient, as ~70% of the recovered streptomycin-resistant colonies contained the desired reversions of the *cj0596 *mutation.

### Mutation of *cj0596 *causes a moderate growth defect of *C. jejuni *in MH broth

**Figure 5:**
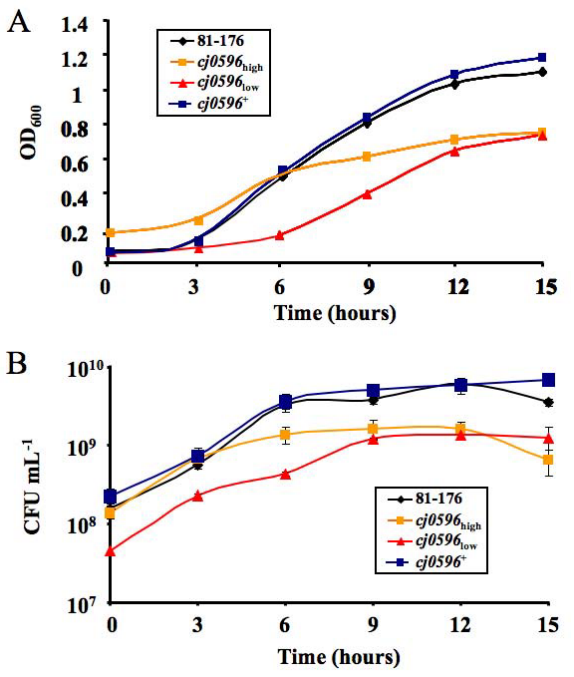
**Growth of *C. jejuni *strains at 37°C in MH broth**. Strains 81–176 (black diamonds), 81–176*cj0596 *("low" inoculum, red triangles) and 81–176*cj0596*^+ ^(blue squares) were inoculated at an OD_600 _of ~0.06 and growth was monitored by OD_600 _(A) as well as CFU mL^-1 ^(B). Additionally, 81–176*cj0596 *("high" inoculum, orange squares) was inoculated at an OD_600 _of ~0.2.

The role of Cj0596 in *Campylobacter *growth was studied by comparing the growth rates of *C. jejuni *81–176, 81–176*cj0596*, and 81–176*cj0596*^+ ^in MH broth (Figure 5). When inoculated at OD_600 _~ 0.06 and growth rate was measured by OD_600 _(Figure 5A), the mutant initially appeared to have a significant growth defect illustrated both by a slower increase in OD_600 _and a lower maximum OD_600_. However, we also found that a similar starting OD_600 _resulted in a significantly lower starting CFU for the mutant, indicating that the OD_600 _did not accurately reflect the number of *cj0596 *mutant CFU. We then grew the mutant under two starting conditions: one had the same starting OD_600 _as the wild-type and revertant (OD_600 _~ 0.06) and the other had approximately the same starting CFU (OD_600 _~ 0.2) as the wild-type and the revertant. The mutant inoculated at OD_600 _~ 0.2 showed a faster initial increase in OD_600_, but reached a similar maximum OD_600 _as the mutant inoculated at OD_600 _~ 0.06. However, when growth rate was monitored by plating for viable counts (Figure 5B), the mutant had an initial growth rate more similar to that of wild-type, although both mutant cultures yielded final viable counts lower than wild-type. Wild-type growth characteristics were restored in the revertant strain. Together, these data suggest that mutation of *cj0596 *resulted in a moderate growth defect, especially later in the growth curve, and that the *cj0596 *mutant had apparent changes in cell characteristics such that the OD_600 _had poor concordance with CFU measurements.

### Deletion of *cj0596 *increases the motility of *C. jejuni*

**Figure 6:**
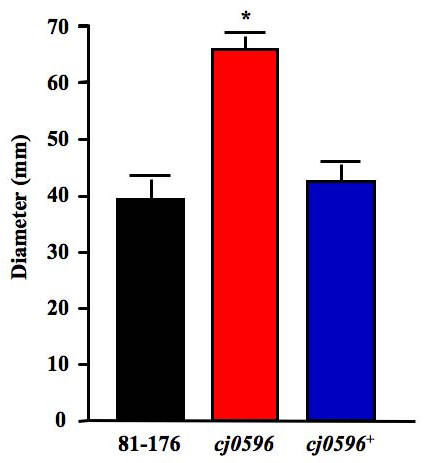
**Motility of *C. jejuni *strains at 37°C**. MH motility plates (0.4% agar) were inoculated with strains 81–176 (black), 81–176*cj0596 *(red) and 81–176*cj0596*^+ ^(blue) and the zones of motility were measured after 48 hours. Statistical significance (*p *< 0.05) is represented by an asterisk.

Because motility plays an important role in invasion of host intestinal cells and is required for animal colonization, the motility of *C. jejuni *81–176, 81–176*cj0596*, and 81–176*cj0596*^+ ^was compared at 37°C (Figure 6). The average diameter of the zone of motility for the wild-type was 39.3 mm ± 3.7 at 48 h. The mutant was significantly more motile with a zone diameter of 66.0 mm ± 2.4 (p < 0.0001). The revertant returned to wild-type motility levels with a zone diameter of 42.5 mm ± 3.0. A similar increase in motility was seen when the assay was performed at 42°C (data not shown). Thus, Cj0596 is involved in the expression of motility.

### Deletion of *cj0596 *increases the ability of *C. jejuni *to invade INT407 cells, but does not affect adherence or intracellular survival

**Figure 7:**
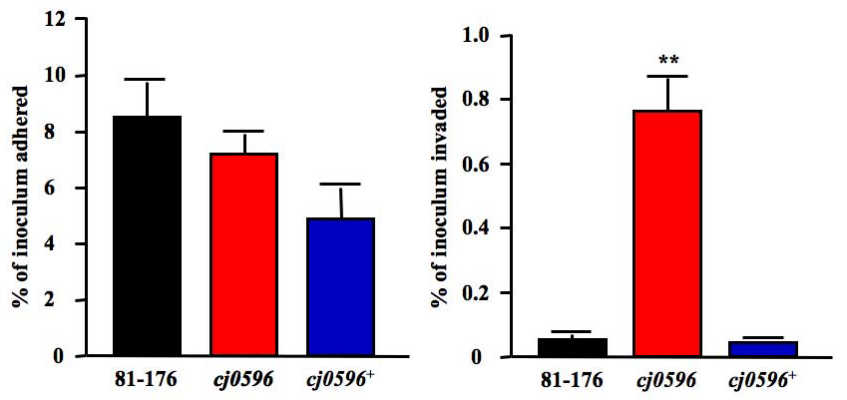
**Abilities of *C. jejuni *strains to adhere to and invade INT407 cells**. Strains 81–176 (black), 81–176*cj0596 *(red) and 81–176*cj0596*^+ ^(blue) were grown to mid-log phase in biphasic culture. INT407 monolayers were inoculated with bacteria at an MOI of ~40. After 3 h, the cells were washed and bacteria adhered were enumerated (A). Gentamicin was added to another plate of cells and incubation was continued for an additional 2 h after which the cells were washed and bacteria invaded were enumerated (B). Statistical significance (*p *< 0.001) is represented by two asterisks.

The possibility that Cj0596 plays a role in interaction with host cells was studied by comparing the adherence and invasion abilities of *C. jejuni *81–176, 81–176*cj0596*, and 81–176*cj0596*^+ ^in an *in vitro *assay using INT407 intestinal epithelial cells (Figure 7). The mean percentages of the inoculum that adhered were 8.5 (± 1.4), 7.2 (± 0.7), and 4.7 (± 1.2) for the wild-type, mutant, and revertant, respectively, demonstrating that deletion of Cj0596 does not significantly affect the ability of *C. jejuni *to adhere to INT407 cells (p > 0.05; Figure 7A). In contrast, mutation of *cj0596 *had a significant effect on the invasion ability of *C. jejuni*. While the percentages of the wild-type and revertant inocula invading INT407 cells were 0.041 (± 0.007) and 0.027 (± 0.005), respectively, the *cj0596 *mutant showed a nearly 20-fold increase in invasion (0.76 ± 0.11, p < 0.001; Figure 7B). The gentamicin and Triton X-100 sensitivities of the three strains were tested to ensure that the invasion results were not due to altered killing of a strain, and no significant difference was found for either compound.

Compared to the number of bacteria that invaded the INT407 cells, the percentages of wild-type, mutant, or revertant that were able to survive intracellularly at timepoints from 2 to 16 hours post-invasion were not significantly different (data not shown).

### 81–176*cj0596 *is defective in mouse colonization

**Figure 8:**
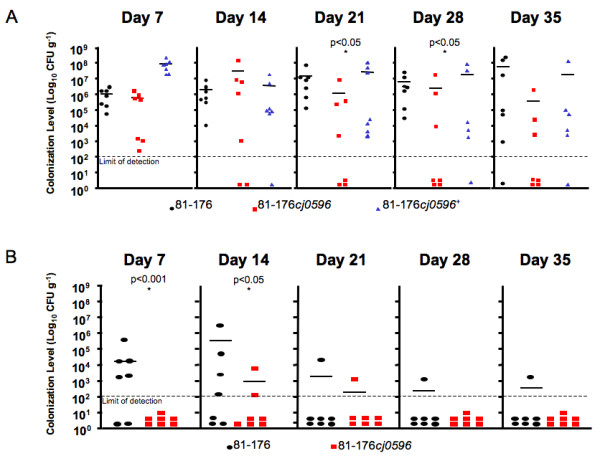
**Colonization of BALB/c-ByJ mice by *C. jejuni *strains**. The abilities of strains 81–176 (black circles), 81–176*cj0596 *(red squares), 81–176*cj0596*^+ ^(blue triangles) to colonize BALB/c-ByJ mice alone (A) and in competition (81–176 [black circles], 81–176*cj0596 *[red squares]) (B) were measured. Mice were fed 1 × 10^9 ^CFU of each strain, or a mixture of 81–176 and 81–176*cj0596 *(5 × 10^8 ^CFU each) by oral gavage. Colonization levels were measured by enumeration of bacteria present in fecal pellets on days 7, 14, 21, 28, and 35 post-inoculation.

To determine whether Cj0596 plays a role in mouse colonization, we used a BALB/c model that has been used previously to assess colonization differences between wild-type and mutant bacteria [34,57,67]. Female BALB/c-ByJ mice were given doses of *C. jejuni *81–176, 81–176*cj0596*, and 81–176*cj0596*^+ ^individually (1 × 10^9 ^CFU each), as well as a mixture of wild-type and *cj0596 *mutant (5 × 10^8 ^CFU each) in a competition experiment, and colonization was measured by determining viable counts of bacteria in fecal pellets at weekly intervals (Figure 8).

On days 7 and 14, viable bacteria were found in all seven mice receiving the wild-type, mutant, or the revertant (Figure 8A). Following the peak in colonization at 14 days, viable mutant bacteria were recovered from only four mice on day 21, and only three mice on days 28 and 35. At these latter three timepoints, the wild-type and revertant were recovered from all but one mouse. The mean colonization densities of the wild-type and revertant were 1.0 × 10^6 ^and 8.4 × 10^7 ^CFU/g, respectively, on day 7 and remained relatively consistent throughout the experiment. The mean colonization level of the mutant was significantly lower than wild-type and revertant on days 21 (1.51 × 10^5 ^CFU/g; p < 0.05) and 28 (3.42 × 10^6^CFU/g; p < 0.05). When placed in competition with the wild-type, the mutant showed an inability to compete for colonization (Figure 8B). Wild-type bacteria were recovered from five mice on day 7, four mice on day 14, and then one mouse for the remainder of the experiment. Viable mutant bacteria were recovered from no mice on day 7 (p < 0.001), two mice on day 14 (p < 0.05; the peak in colonization, as observed in mice given the mutant alone), one mouse on day 21, and then were not recovered on days 28 and 35.

### Deletion of *cj0596 *alters *C. jejuni *protein expression

**Figure 9:**
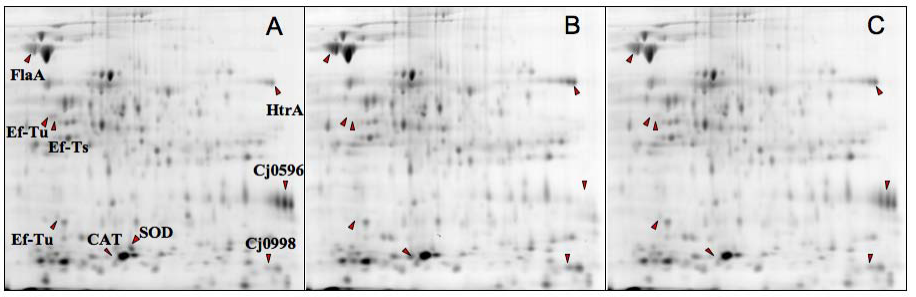
**Differences in protein expression in *C. jejuni *strains**. 2-D SDS-PAGE gels (12%) showing: wild-type (A), *cj0596 *mutant (B), and *cj0596 *revertant (C) protein profiles. Proteins with greater expression in *cj0596 *mutant (fold change): HtrA (+2.5), Cj0998 (+2.1), FlaA (+2.0). Proteins with lesser expression in *cj0596 *mutant (fold change): EF-Ts (-2.9), SOD (-2.6), EF-Tu (-2.0, -1.9). CAT was found only in the *cj0596 *mutant, and Cj0596 was absent in the mutant. Each of these protein expression differences returned to a level statistically similar to wild-type in the revertant.

Because Cj0596 is thought to be a periplasmic chaperone, its loss could result in compensatory changes in the expression of other proteins. To determine the effect that deletion of Cj0596 had on the expression of other proteins, a comparison of total cell proteins from *C. jejuni *81–176, 81–176*cj0596*, and 81–176*cj0596*^+ ^was performed using 2D SDS-PAGE (Figure 9). Three proteins were found to be significantly upregulated in the mutant. They were identified as HtrA (2.5-fold), Cj0998 (2.1-fold), and FlaA (2.0-fold). As expected, the CAT protein was found only in the Cj0596 mutant. Conversely, the Cj0596 protein was found in wild-type and revertant strains, but was absent in the *cj0596 *mutant, as expected. Three proteins were found to be significantly downregulated in the mutant. These proteins were EF-Ts (2.9-fold), superoxide dismutase (SOD) (2.6-fold), and EF-Tu (two spots; 2.0-fold, 1.9-fold). All of the proteins that showed altered abundance in the mutant returned to near wild-type levels in the revertant.

## Discussion

*C. jejuni *is a major cause of human diarrheal infection worldwide, yet we have only limited knowledge regarding the mechanisms the bacterium uses to colonize humans and cause disease. Because *C. jejuni *inhabits two hosts with differing body temperatures, we became interested in proteins (including Cj0596) that are more abundant when *C. jejuni *is grown at 37°C (human body temperature) compared to 42°C (chicken body temperature). Because of its homology to other PPIases that are involved in the virulence of other bacteria and the fact that it is highly conserved among *Campylobacter *species, this protein may play an important role in human colonization. *In silico *analyses of the gene and protein sequences suggest that Cj0596 is probably a periplasmic PPIase that is involved in folding integral outer membrane proteins.

Among the changes that occur in bacterial cells when encountering lower growth temperatures are a decrease in membrane fluidity, and inefficient folding of some proteins [68]. Proper protein folding or refolding of cold-damaged proteins is important after cold shock, and certain chaperones may be upregulated during cold shock in an attempt to compensate for the decreased efficiency of protein folding [69]. In *E. coli*, several molecular chaperones (including GroEL, GroES, *htpG*, *ppiA*, and trigger factor) were transiently induced upon cold shock [69,70]. Additionally, the chaperone ClpB may renature and solubilize aggregated proteins at low temperatures at which translation is repressed [71]. Whether the modest induction of Cj0596 that seems to occur at 37°C has any biological relevance to protein folding in the periplasm or modulation of membrane fluidity is a topic for future study.

Cj0596 is similar to the *E. coli *protein SurA, which is a peptidyl prolyl *cis-trans *isomerase located in the periplasm and which plays a role in folding outer membrane proteins, particularly LamB and OmpA, and in pilus biogenesis [72-74]. A UPEC strain in which SurA was inactivated was less able to bind and invade bladder epithelial cells, in addition to showing a decreased ability to survive intracellularly [75].

There are several other examples of PPIases, and SurA orthologs in particular, having roles in bacterial pathogenesis. In *S. flexneri*, SurA is required for proper folding and insertion into the outer membrane of IcsA, which is responsible for the ability of the bacterium to spread intercellularly [28]. Deletion of SurA decreases the ability of *S. enterica *to adhere to and invade Caco-2 and RAW264.7 cells *in vitro*, as well as reducing the capacity to colonize BALB/c mice [24]. A *L. pneumophila *mutant lacking the PPIase Mip was defective in initiating macrophage infection *in vitro*, and less virulent when introduced into a guinea pig model [23]. Similarly, the *C. trachomatis *Mip-like protein and the *T. cruzi *TcMip protein play roles in the early steps of intracellular infection by these bacteria [26,27]. Ng-MIP, found in *N. gonorrhoeae*, is similar to these Mips, but plays a role in intracellular survival rather than invasion [25].

Previously, a *C. jejuni *NCTC 11168 *cj0596 *mutant was found to have a decreased growth rate when growth was measured by OD_600 _[29]. Our measurements by OD_600 _initially suggested that the mutant had a reduced growth rate, but when growth was monitored using viable counts, the mutant was found to grow initially at a rate more similar to the wild-type, although a modest growth defect was still apparent at later stages of growth (Figure 5). The difference in the results obtained by OD_600 _and viable counts might be the result of a change in cell size or the light scattering properties of the *cj0596 *mutant, possibly caused by a change in the composition of the outer membrane of the bacterium. Future work, such as using electron microscopy to evaluate the shape and surface components of the mutant, might help explain the reason for the discrepancy between the results obtained by OD_600 _measurements and viable counts.

*C. jejuni *has two polar flagella which play a major role in virulence. Flagella-mediated motility is responsible for colonization of the mucous lining of the mammalian and avian gastrointestinal tracts as well as invasion of gastrointestinal epithelial cells [55,76-78]. We found that the *cj0596 *mutant was significantly more motile than wild-type bacteria. Because we found that removal of Cj0596 increased the motility of the bacterium, we considered that the *cj0596 *mutant might be more invasive than wild-type. Studies using INT407 cells showed that mutation of *cj0596 *did in fact increase the invasiveness of *C. jejuni *without altering the adherence and intracellular survival abilities. Previously, it was noted that a *C. jejuni *NCTC 11168 *cj0596 *mutant was significantly deficient in its ability to adhere to host cells [29]. The discrepancy in adherence results seen between the previous study and our current work could be due to strain differences, however, we cannot exclude the possibility that the previously obtained adherence phenotype was due to an unlinked mutation in the uncomplemented NCTC 11168 *cj0596 *mutant. The increased motility and invasiveness could be due to an increase in chemotaxis, or to increased flagellar function because of a change in outer membrane architecture or cell morphology that provides a motility advantage.

Several proteins located on the cell surface play a role in the initial cell-to-cell contact that is a component of intestinal colonization by *C. jejuni*. Because Cj0596 is thought to be involved in folding outer membrane proteins, its mutation is likely to have an effect on surface-exposed proteins, which could affect the ability to colonize the host intestinal tract. When mice were inoculated individually with the wild-type, mutant, or revertant, the *cj0596 *mutant initially was able to colonize at mean levels comparable to the wild-type and revertant strains. However, the mutant became increasingly colonization deficient over time. The differences were statistically significant at days 21 and 28, but not at day 35 due to increased clearance of the wild-type and revertant strains from some mice. This colonization defect is likely not the result of the increased motility of the mutant, since motility typically correlates with better animal colonization. One possible explanation for the decreased colonization ability of the mutant is that Cj0596 is required for the proper presentation of surface structures that are necessary for mouse colonization (e.g., known or unknown adhesins, oxidative stress, or other mouse colonization factors). Additionally, when the mutant was placed in direct competition with the wild-type, it demonstrated an inability to compete with the wild-type for colonization of the mice. In competition experiments, curiously, colonization levels of both the wild-type and mutant were significantly lower (compared to individual infections), suggesting some sort of interference of these strains with each other. The *cj0596 *mutant shows elevated autoaggregation and biofilm formation (manuscript submitted), so it is possible that these or other features impacting *C. jejuni *community structure could be involved.

In an effort to determine some of the molecular causes of the altered virulence phenotypes discussed previously, we conducted a proteomic analysis comparing the whole-cell protein profiles of wild-type, mutant, and revertant. As expected, CAT was found only in the mutant and Cj0596 was absent in the mutant, confirming the replacement of *cj0596 *with the *cat *cassette in the mutant, and restoration of Cj0596 expression in the revertant.

A total of six other proteins showed altered expression in the *cj0596 *mutant. All proteins that showed altered abundance in the mutant returned to near wild-type levels in the revertant. Three proteins were found to be significantly upregulated in the mutant. They were identified as HtrA (2.5-fold), Cj0998 (2.1-fold), and FlaA (2.0-fold). HtrA is a serine protease with homologs found in most bacteria. In *E. coli*, HtrA is located on the periplasmic side of the inner membrane [79,80], has protease activity [81], and has some chaperone activity at low temperatures [82]. It is possible that *C. jejuni *HtrA is upregulated in the mutant in a compensatory manner due to an increase in unfolded protein in the periplasm resulting from the loss of a major periplasmic PPIase. Brondsted *et al*. [83] found that a *C. jejuni *HtrA mutant showed no change in motility or autoagglutination, but did have a decreased ability to adhere to and invade INT407 cells and also exhibited altered cell morphology. Cj0998 is annotated as 'putative periplasmic protein' and is restricted primarily to the epsilon proteobacteria, although the function of this protein is currently unknown. FlaA is the major subunit of the *C. jejuni *flagellum, and the upregulation of FlaA is consistent with the increase in motility and invasion of INT407 cells seen in the *cj0596 *mutant.

Three proteins were shown to be significantly downregulated in the *cj0596 *mutant. They were identified as EF-Ts (2.9-fold), SOD (2.6-fold), and EF-Tu (two spots; 2.0-fold, 1.9-fold). Among proteins whose expression was lower in the *cj0596 *mutant, EF-Ts and EF-Tu are involved in protein translation. They may be downregulated in the mutant due to an increase in unfolded protein in the periplasm and this in turn may result in the late stage growth defect due to a general decrease in protein synthesis. As *C. jejuni *lacks a sigma-E response [22], the signalling mechanism that would be responsible is unknown. SOD plays a role in protecting *C. jejuni *against damage from oxidative stress and mutation of *sod *in *C. coli *was found to decrease the ability of the bacterium to colonize the intestines of 1-day-old chicks [84]. The decreased levels of SOD in the *cj0596 *mutant may therefore play a role in the colonization defects seen in mice.

## Conclusion

Cj0596 is a highly conserved protein whose expression in *C. jejuni *is induced at human body temperature. Bacteria lacking Cj0596 were found to exhibit changes in several virulence-related phenotypes, including motility and host cell invasion, as well as alterations in protein expression and a defect in mouse colonization.

## Authors' contributions

JEH carried out the proteomics experiments comparing 81–176 grown at 37°C and 42°C. KMR carried out all other experiments and participated in the study design and drafting of the manuscript. SAT conceived the study and participated in the study design and drafting of the manuscript. All authors read and approved the final manuscript.
